# Sleep duration and perceptions of sleep quality in British Army recruits during basic training - an observational analysis

**DOI:** 10.3389/fneur.2024.1321032

**Published:** 2024-02-14

**Authors:** Alex J. Rawcliffe, Hayley Tyson, Katrina Hinde, Kimberley Jacka, Rachel Holland, Shaun Chapman, Andrew J. Roberts

**Affiliations:** ^1^Army Recruit Health and Performance Research, Medical Branch, HQ Army Recruiting and Initial Training Command, Ministry of Defence, Upavon, United Kingdom; ^2^Faculty of Science and Engineering, Anglia Ruskin University, Cambridge, United Kingdom; ^3^Human Sciences Group, Defence Science and Technology Laboratory, Salisbury, United Kingdom; ^4^Cambridge Centre for Sport and Exercise Sciences, School of Psychology and Sport Science, Anglia Ruskin University, Cambridge, United Kingdom

**Keywords:** recruit, sleep duration, sleep loss, military training, adolescence

## Abstract

**Introduction:**

Sleep is critical to the health, wellbeing and performance of military personnel during basic training. This two-part study evaluated sleep-wake patterns and sleep disturbances in junior soldiers (JS) and infantry recruits in Autumn 2021 (study 1), and non-infantry recruits in spring 2022 (study 2).

**Methods:**

During studies 1 and 2, validated wearable technology combined with a sleep diary was used to quantify sleep-wake indices, sleep disturbances and perceptions of sleep quality. Sleep diary data was analysed descriptively. A series of repeated-measures ANOVAs examined differences in objective sleep-wake indices. Correlation analysis determined associations between time in bed (TIB) and total sleep time (TST).

**Results:**

Significant (*p* < 0.05) differences in most sleep-wake indices were observed between weeks of basic training for all cohorts. Strong positive correlations between TIB and TST were observed for each cohort across basic training (*r* = 0.681 – 0.970, *p* < 0.001), with longer TST associated with greater TIB. The mean±SD sleep duration (hours and mins [hm]) for JS (06:22 ± 00:27hm), non-infantry (05:41 ± 00:47hm) and infantry (05:46 ± 00:34hm) recruits across basic training was consistently below national recommendations. The mean±SD bed and wake times for JS (bedtime: 23:01 ± 00:32hm; awake: 05:34 ± 00:10hm), non-infantry (bedtime: 23:38 ± 01:09hm; awake: 04:47 ± 00:58hm), and infantry (bedtime: 23:13 ± 00:29hm; awake: 05:38 ± 00:26hm) recruits varied across weeks of basic training, with over 80% reporting “fairly bad” or “very bad” sleep quality and frequent periods of “dozing off” during daytime activity. The most commonly reported sleep disturbing factors identified during basic training involved: late-night military admin (e.g., ironing, boot cleaning, kit set up etc), early morning wake times, extraneous noise, light and hot room temperatures within the primary sleeping environment, bed/mattress discomfort, muscle soreness and feelings of stress and anxiety.

**Discussion/Conclusion:**

Our findings contribute to the existing evidence that long-term sleep loss is pervasive during initial military training programmes. The average sleep durations indicate chronic and unrecoverable sleep loss which would be expected to significantly impair physical and cognitive military performance, and increase the risk of injury, illness and attrition rates during basic training. Changes in the design and scheduling of basic training programmes to enable, at the least, minimum sleep recommendations to be met, and to improve sleep hygiene in the primary sleeping environment are warranted.

## Introduction

The primary aim of basic training is to transform civilians into trained soldiers. Recruits are required to internalize core professional values, master technical skills and improve physical fitness to meet the required standards of basic training. Failure to meet these standards can negatively impact first time pass rates and subsequent progression into the field Army as a fully trained soldier. Poor health and performance during basic training has been associated with a number of risk factors (e.g., low fitness, prior injury, cognitive dissonance and stress, aggressive coping strategies, poor mental health, smoking, body mass index) (1–4). However, a component of basic training that serves to promote several physiological and cognitive functions, and therefore, could significantly undermine recruit health and performance during basic training if impaired, is sleep.

Good quality sleep is dependent on achieving sufficient duration and is essential for recovery from training and operational stressors (5). The negative implications of poor sleep on mental health (6, 7), immune function and infection (8), physical adaptation and performance (9), injury risk (10, 11), emotional regulation (12), cognitive and higher-order functioning (13), and job performance (14) are well established in civilians (both adult and adolescent) and military personnel. However, despite a growing awareness of the importance of sleep, military culture largely accepts sleep deprivation as a normal part of military training, with reports indicating that military leaders and, by extension, their subordinates perceive the need for sleep as a “weakness” (15, 16) or as a means of “hardening” recruits as part of their socialization into the military (17). These actions are despite prior research demonstrating the importance of leadership’s role in subordinates’ sleep health, particularly recruits who include the youngest and least experienced members of the military (17, 18).

According to expert consensus statements (19) and the National Sleep Foundation (NSF) recommendations for sleep duration (20), healthy young adults [18–25 years (yrs)] and adolescents (13-18 yrs) (representative of recruits and Junior Soldiers [JS] undergoing regular basic training) require 7–9 h (hr) and 8–10 h of sleep per night to support optimal health and performance, respectively. Despite these recommendations, it is becoming increasingly evident that military recruits are likely to experience chronic and unrecoverable sleep loss during basic training (21–23), and therefore, placing them at greater risk of injury, illness, and impaired cognitive and military specific performance (10, 13, 24). Evidence of inadequate sleep duration in British Army Officer Cadets (5.5 h per night) and Infantry recruits (6 h per night) has been reported (8, 25). However, these studies only assessed a small number of days (7–14) relative to training course length (14–40 weeks), did not include an assessment of sleep hygiene of the primary sleeping environment, and were unable to evaluate sleep from other basic training populations (e.g., non-infantry or junior soldiers).

To date, no assessment of sleep–wake patterns of non-infantry recruits and JS during basic training has been conducted. The average age of a JS commencing basic training is 16 yrs., and of the many changes that occur during maturation, changes in the adolescent sleep–wake cycle are among the most dramatic (26–28). Coinciding with pubertal onset and throughout maturation, adolescents experience a phase delay (i.e., a shift in chronotype preference) in their sleep–wake cycle as reflected by delayed bedtimes, later awakenings and longer sleep periods (27, 29, 30). These biologically determined adaptations in adolescent sleep–wake patterns dictate a net increase of 0.5–1.25 h, which corresponds to 8.5–9.25 h of required sleep per night, irrespective of adolescent age or maturation stage (17, 31). Poor understanding of the sleep–wake changes and requirements of adolescents combined with chronic and unrecoverable sleep loss risks the presentation of significant health, performance and developmental issues during maturation (29, 32). As such, better understanding of the current sleep–wake patterns of JS during training is warranted given the health and performance implications related to poor and unrecoverable sleep loss as demonstrated within civilian populations (30).

Our current understanding of sleep across different training populations is limited, and therefore, unable to adequately support evidence-based practice/policy changes related to sleep and recovery of recruits and JS during basic training. The aim of this study was to evaluate sleep–wake patterns and perceptions of sleep quality, including potential sleep hygiene issues, in JS, non-infantry and infantry recruits during their respective basic training. We hypothesized that all recruits and JS would demonstrate inadequate sleep duration relative to minimum national sleep recommendations, poor perceptions of sleep quality, and suboptimal sleep hygiene within the primary sleeping environment.

## Methods

Study 1 and 2 conducted an assessment of sleep duration and perceptions of sleep quality during basic training. Study 1, conducted in autumn 2021, used wrist-based actigraphy (wGT3X-BT, Actigraph, Pensacola, United States) and the National Sleep Foundation (NSF) sleep diary during weeks 1, 2, 6 and 11 of basic training in line infantry recruits (Infantry Training Center, Catterick) and Junior Soldiers (JS) (Army Foundation College, Harrogate). Study 2, conducted in spring 2022 used a sleep ring (Oura health, Finland) and the same NSF sleep diary during 12 weeks of basic training in non-infantry recruits (Army Training Center, Pirbright). To note, study 2 was part of a wider internal service evaluation on program design, explaining why the sample size for this group was greater compared to JS and infantry recruits. Participants who volunteered to take part in this study provided informed consent. Ethical approval was granted by the United Kingdom Ministry of Defense Research Ethics Committee (924/MODREC/18). Height and weight were recorded using a stadiometer and digital weighing scales (SECA 703, Birmingham, United Kingdom). Body mass index was calculated and interpreted as per Nuttall (33) (Table 1).

**Table 1:** Participant demographics.

Basic training cohort	Age (yrs)	Height (cm)	Body mass (kg)	BMI (kg/m^2^)
Non-infantry recruits (*n* 208)	23.2 ± 4.8	175.9 ± 8.4	75.8 ± 5.4	24.2 ± 4.1
Junior Soldiers (*n* 37)	16.2 ± 0.4	164.4 ± 35.1	71.7 ± 10.7	24.5 ± 3.1
Line Infantry recruits (*n* 19)	22.8 ± 4.4	175.7 ± 8.5	73.2 ± 9.6	23.8 ± 3.2

Values are mean ± SD.

### Experimental design

The sleep–wake indices recorded from the wearable technology during both study 1 and 2 are defined in Table 2 (34, 35).

**Table 2:** Sleep–wake indices.

Sleep variable	Definition (units)
Total sleep time	Actual time spent asleep from sleep start and end, minus any wake time (hours: mins [hm])
Time in bed	Difference between bedtime and awakening time (hm)
Sleep onset latency	The time it takes to transition from full wakefulness to sleep onset (hm)
Wake after sleep onset	The total number of hours and mins marked awake after sleep onset (hm)
Sleep efficiency	The sleep duration expressed as a percentage of time asleep from bedtime to wake (%)
Sleep fragmentation index	A measure of restlessness based on physical movement and expressed as a percentage (%)

A description of the sleep indices collected from the wearable technology used in both study 1 and 2.

### Study 1

The Actigraph wGT3X+ sleep watch (dimensions: 4.6 cm x 3.3 cm x 1.5 cm, weight: 19 g) was worn on the non-dominant wrist and used to record sleep–wake indices from infantry recruits and JS. The wGT3X+ has shown to produce valid estimates of sleep–wake indices when worn on the wrist compared to polysomnography (PSG) (36). Due to limited resources at the time of data collection, sleep–wake data was only measured (Monday to Friday) during weeks 1, 2, 6 and 11 of basic training. Actigraph watches were initialized to record sleep–wake data in 30-s epochs, with mean ± SD values reported across each week of basic training. Sleep–wake data was derived from proprietary software (ActiLife Software, v6.13.4, Pensacola, FL) using the Sadeh algorithm (37), which has shown reasonable to good levels of agreement for sleep–wake estimation compared to PSG (38). An online version (LimeSurvey, Community Edition Version 6.3.4) of the NSF sleep diary (completed on individual’s mobile phones) was used to determine perceptions of sleep quality and sleep disturbing factors, involving a series of multiple-choice questions, including “*likeliness of dozing off during daytime*,” “*ease of falling asleep at night,”* “*overall rating of sleep quality*,” “*perceived fatigue upon awakening*,” and *“factors disturbing sleep at night.”* In both study 1 and 2, participants were provided with verbal instructions of the timing (i.e., AM between 0600–0700), how to complete the self-report measures, and were given example question/answer definitions to aid their understanding and interpretation.

### Study 2

A sleep ring (OURA Gen 2), width: 7.9 mm; thickness: 2.6 mm; weight: 4-6 g was worn on the non-dominant index finger and used to record the same sleep–wake indices in study 1 each night of the 12-week course (~90 days). Estimates of sleep staging were not included due to low levels of accuracy and sensitivity reported for the Gen 2 ring when detecting rapid-eye-movement (REM) and non-REM sleep compared to gold standard (35, 39). Data from the sleep ring was extracted from proprietary software (Oura Teams, Finland) and mean ± SD reported across each week of basic training. Compared to gold-standard (i.e., PSG), the sleep ring demonstrates acceptable-to-high levels of accuracy and sensitivity in detecting sleep–wake indices and differences in sleep patterns in young healthy populations (35). The same online NSF sleep diary questions were used to determine perceptions of sleep quality and sleep disturbing factors only in weeks 1, 2, 6 and 12 to minimize study burden while providing comparable data.

### Statistical analysis

Data was analyzed descriptively (mean ± SD) to summarize participants’ demographics, objective sleep–wake indices and scores for each subjective response across the reporting weeks of basic training. The daily TST data derived from the sleep watch for infantry recruits and JS, and the sleep ring for non-infantry recruits were plotted against the NSF recommendations for young adults (18-25 yrs., i.e., non-infantry and infantry recruits) and adolescents (13-18 yrs., i.e., JS) (40). The R package ‘vioplot’ (Alder and Kelly, 2020) (V.0.3.6) in R Core Team software (V.4.0.4; Vienna, Austria) was used for preparation of the TST figures.

Sleep–wake variables collected from the sleep watch and ring were graphically examined for normality (normal Q-Q plots) prior to statistical analysis. Sample size was based on *a priori* power analysis using G*power (Dusseldorf, V 3.1). For a within-factors repeated-measures (RM) ANOVA, a minimum of 18 participants was required to achieve a medium effect size (np^2^, 0.06) with *α* = 0.05 and *β* = 0.90. A series of RM ANOVAs were conducted to determine differences in pseudo-objective sleep–wake indices between each week of training measured. Significant main effects were followed up with *post hoc* (Bonferroni adjusted) analyses and mean differences between significant pairs were presented with corresponding effect sizes [Cohen’s *d* criteria: ≤ 0.2 is considered trivial, 0.21–0.5 is small, 0.51–0.8 is moderate and ≥ 0.8 is large (41)]. A bivariate Pearson product–moment correction was conducted to determine the association between TIB and TST for each cohort and week of basic training [Pearson’s r criteria: small 0.10–0.29; moderate 0.30–0.49; large 0.50–1.0 (41)].

## Results

### Study 1

#### Infantry recruits

**Figure 1:**
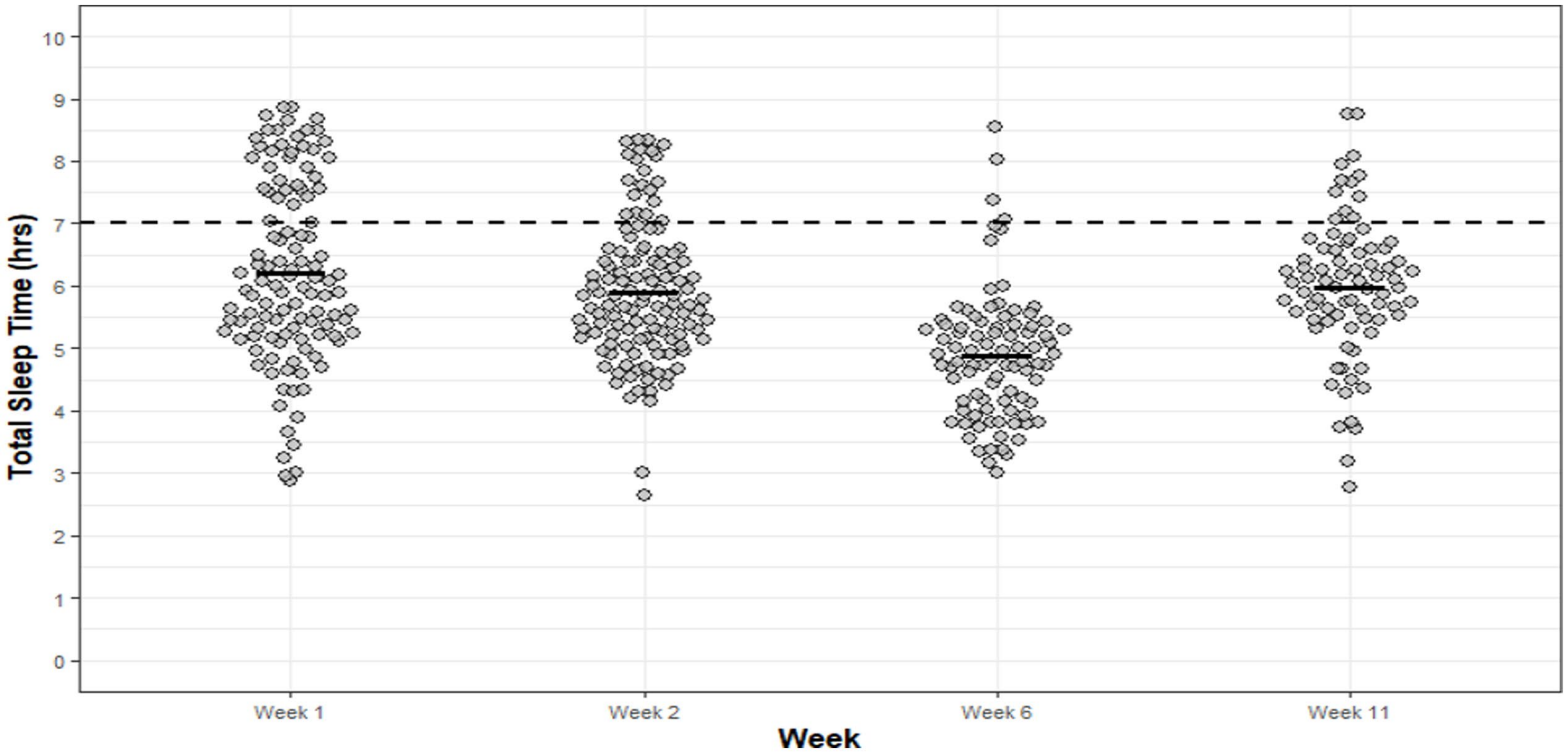
Infantry TST for each night of each week of basic training. Dots are individual nights within their respective week and the solid horizontal line represents the average weekly TST. The dashed horizontal line represents the minimum national sleep recommendations for young adults (7 h).

Significant main effects for week of basic training were observed for sleep efficiency (*p* = 0.017, η_p_^2^ = 0.21), TIB (*p* < 0.001, η_p_^2^ = 0.58), TST (*p* < 0.001, η_p_^2^ = 0.57) and WASO (*p* = 0.005, η_p_^2^ = 0.26). No significant main effects were observed for sleep fragmentation (*p* = 0.420, η_p_^2^ = 0.06) or SOL (*p* = 0.119, η_p_^2^ = 0.102), however, the average sleep fragmentation and SOL was 22 ± 1.5% and 00:07 ± 00.06hm, respectively. *Post hoc* analysis (Supplementary Table S3) revealed significantly poorer sleep efficiency scores in week 1 when compared to week 11 of basic training (*p* = 0.044, 95%CI: 0.06, 5.77, *d* = 1.4, ∆ = 3%). Despite these differences, sleep efficiency remained normal (i.e., >80%) (34) across each week of basic training measured. Infantry recruits had significantly less TIB during week 6 (05:29 ± 00:37hm) when compared to week 1 (*p* < 0.001, 95%CI: 43.11, 145.04, *d* = 2.3, ∆ = 94 min), week 2 (*p* < 0.001, 95%CI: 36.98, 108.79, *d* = 1.8, ∆ = 72 min) and week 11 (*p* < 0.001, 95%CI: 44.52, 103.44, *d* = 1.8, ∆ = 73 min). Similarly, significantly less TST was observed during week 6 (04:48 ± 00:33hm) compared to week 1 (*p* < 0.001, 95%CI: 32.93, 131.87, *d* = 2.0, ∆ = 82 min), week 2 (*p* < 0.001, 95%CI: 38.27, 107.84, *d* = 1.8, ∆ = 73 min) and week 11 (*p* < 0.001, 95%CI: 39.08, 117.21, *d* = 1.9, ∆ = 78 min) of training (Figure 1). Significantly longer WASO was observed in week 1 (00:41 ± 00:10) compared to week 11 (*p* = 0.012, 95%CI: 2.78, 25.90, *d* = 1.4, ∆ = 14 min). A strong positive correlation between TIB and TST was observed for each week of basic training Wk1: *r* = 0.970, *p* < 0.001; Wk2: *r* = 0.915, *p* < 0.001; Wk6: *r* = 0.944, *p* < 0.001; Wk11: *r* = 0.829, *p* < 0.001. Time in bed explained 91–98% of the variance in infantry recruit’s TST during basic training.

Infantry recruits spent, on average, 01:20hm less TIB and 01:17hm less TST during week 6 of basic training when compared to all other reporting weeks. The national minimum sleep recommendations for TST were not met, on average, by 68% in week 1, 84% in week 2, 96% in week 6 and 85% in week 11. Average bed and wake times reflected TST and TIB results: week 1 (bedtime: 23:08 ± 00:33hm; wake-time: 06:12 ± 00:34hm), week 2 (bedtime: 23:13 ± 00:29hm; wake-time: 05:47 ± 00:24hm), week 6 (bedtime: 23:45 ± 00:35hm; wake-time: 05:15 ± 00:16hm) and week 11 (bedtime: 22:47 ± 00:28hm; wake-time: 05:20 ± 00:17hm).

**Figure 2:**
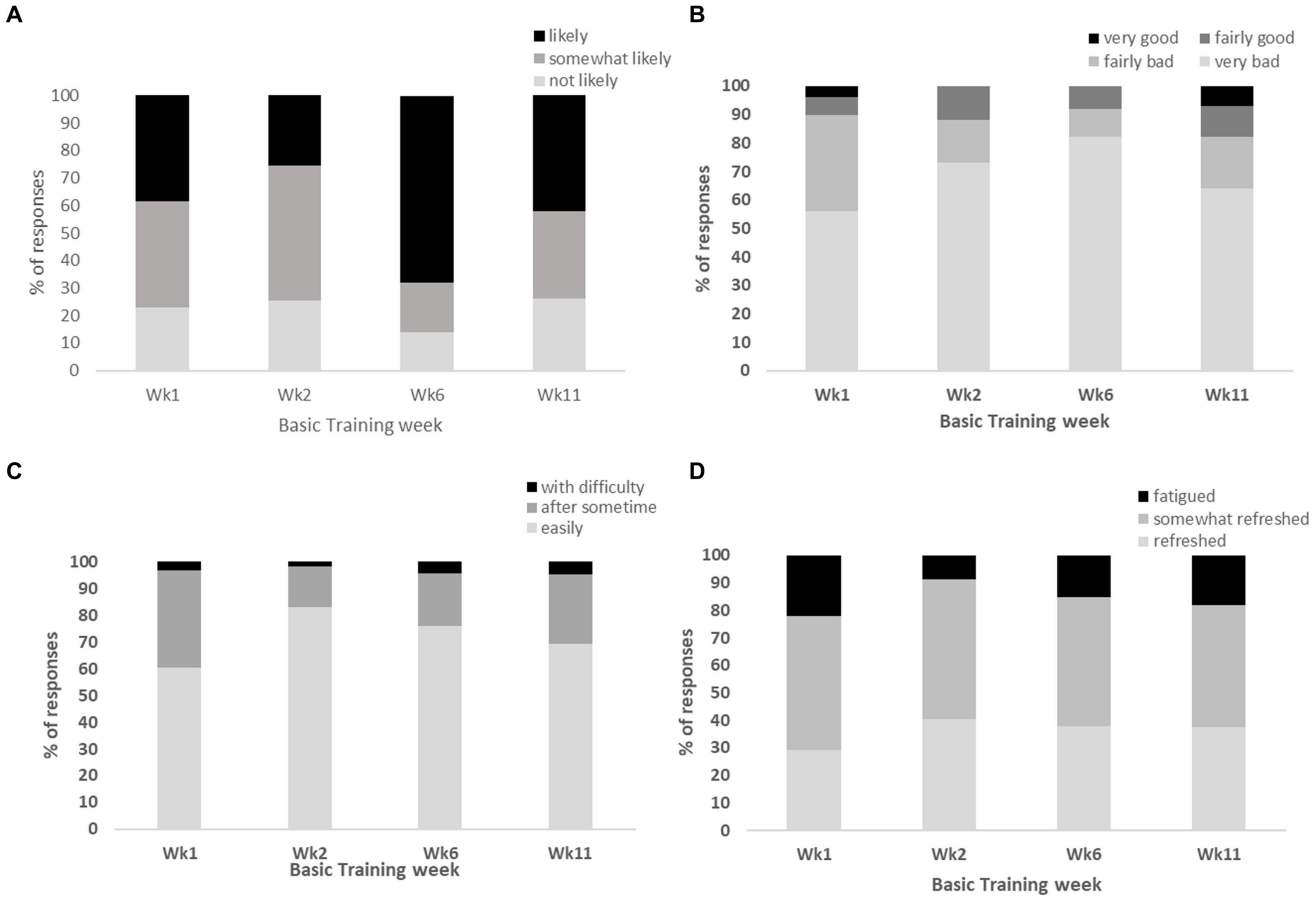
Represents the proportion (%) of responses from infantry recruits for the following NSF sleep diary questions, **(A)** “likeliness of dozing off during daytime”; **(B)** “overall rating of sleep quality”; **(C)** “ease of falling asleep at night”; **(D)** “perceived fatigue upon awakening”.

The NSF sleep diary responses across the reporting weeks of basic training indicated that 38–69% of infantry recruits were “likely” and a further 39–58% “somewhat likely” to doze off during daytime activities. Sleep quality was “very bad” (56–82%) and “fairly bad” (10–34%); while the majority were able to fall asleep “easily” (60–83%) but felt only “somewhat refreshed” (44–59%) upon awakening (Figures 2A–D).

The proportion of the most common self-reported sleep disturbing factors reported by infantry recruits included, late-night military admin (e.g., ironing, boot cleaning, kit set up, weapon handling, studying) and early morning wake times (>85%), noise (36–42%) and light (34–50%) within and outside of the primary sleeping environment, and a hot room temperature (12%).

### Junior soldiers

**Figure 3:**
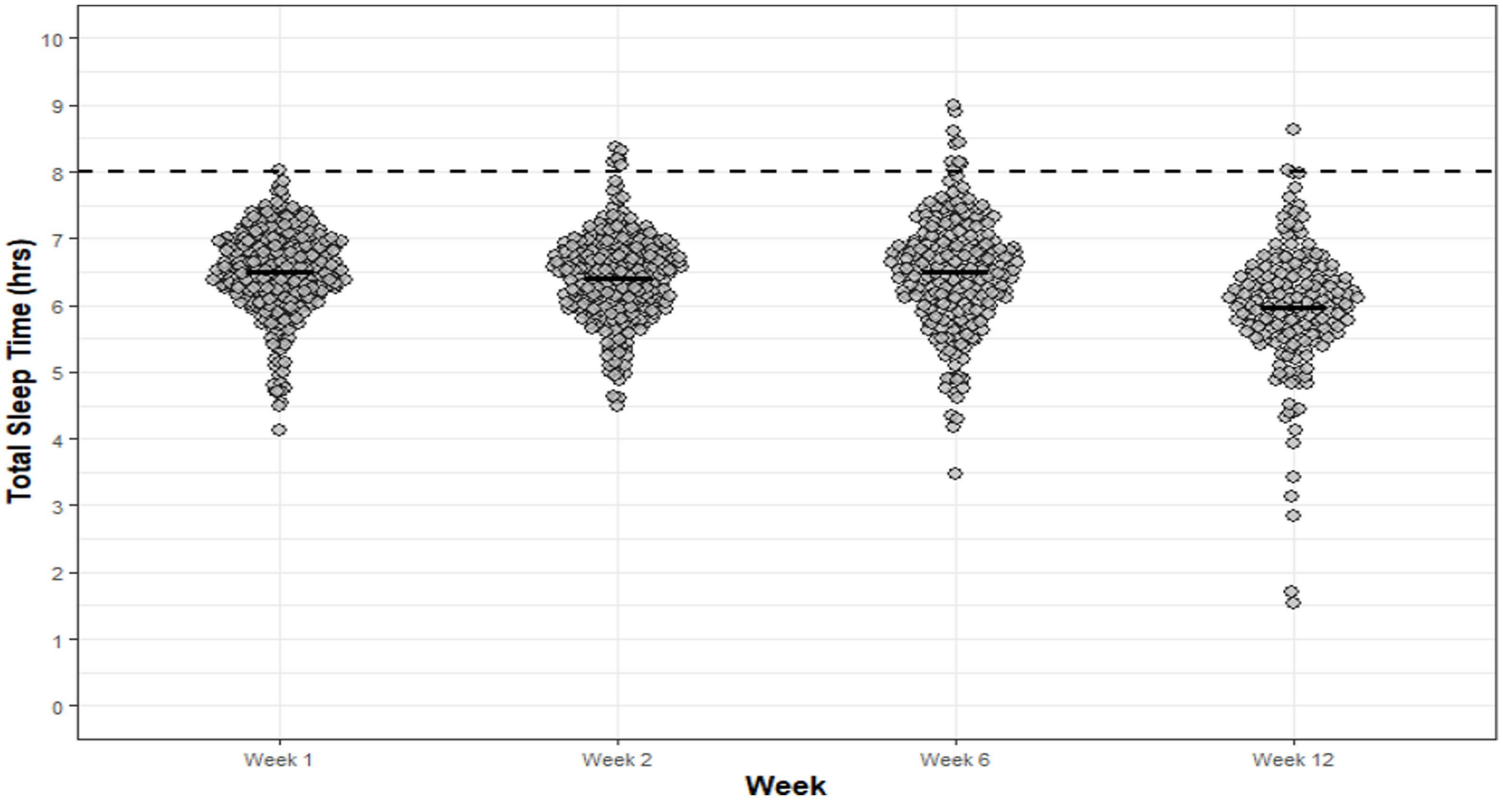
JS TST for each night of each week of basic training. Dots are individual nights within their respective week and the solid horizontal line represents the average weekly TST. The dashed horizontal line represents the minimum national sleep recommendations for adolescents (8 h).

Significant main effects for week of basic training were observed for sleep efficiency (*p* < 0.01, η_p_^2^ = 0.146), TIB (*p* < 0.001, η_p_^2^ = 0.275), TST (*p* < 0.001, η_p_^2^ = 0.319), WASO (*p* = 0.025 η_p_^2^ = 0.080) and sleep fragmentation (*p* = 0.002, η_p_^2^ = 0.130). No significant main effects were observed for SOL (*p* = 0.219, η_p_^2^ = 0.065), with average SOL of 00:14 ± 00:09hm demonstrating a reasonably short SOL throughout basic training. *Post hoc* analysis (Supplementary Table S3) revealed significantly poorer sleep efficiency in week 12 compared to week 1 (*p* = 0.010, 95%CI: −3.84, −0.38, *d* = 0.61, ∆ = 3%) and week 6 (*p* = 0.047, 95%CI: 0.01, 3.31, *d* = 0.47, ∆ = 2%) of basic training. Despite these differences, sleep efficiency was considered normal (i.e., >80%) across the reporting weeks of basic training. Significantly less TIB was observed during week 12 (06:35 ± 00:40hm) compared to week 1 (*p* < 0.01, 95%CI: −44.2, −10.8, *d* = 0.87, ∆ = 27 min), week 2 (*p* = 0.019, 95%CI: −38.2, −2.43, *d* = 0.72, ∆ = 20 min) and week 6 (*p* < 0.01, 95%CI: −47.94, −11.91, *d* = 1.10, ∆ = 26 min). Similarly, significantly less TST was observed in week 12 (06:00 ± 00:43) compared to week 1 (*p* < 0.001, 95%CI: 14.91, 48.8, *d* = 1.02, ∆ = 32 min), week 2 (*p* = 0.004, 95%CI: 5.82, 40.51, *d* = 0.74, ∆ = 23 min) and week 6 (*p* < 0.01, 95%CI: 13.80, 51.23, *d* = 1.04, ∆ = 33 min) (Figure 3). Junior soldiers spent, on average, 00:26hm less TIB and 00:30hm less TST during week 12 when compared to all other weeks evaluated. A strong positive correlation between TST and TIB was observed for each week of basic training Wk1: *r* = 0.809, *p* < 0.001; Wk2: *r* = 0.801, *p* < 0.001; Wk6: *r* = 0.901, *p* < 0.001; Wk12: *r* = 0.949, *p* < 0.001. Time in bed explained 89–97% of the variance in JS TST during basic training.

Junior soldiers demonstrated significantly greater WASO in week 12 (00:33 ± 00:14hm) when compared to week 6 (*p* = 0.017, 95%CI: 0.75, 10.84, *d* = 0.45, ∆ = 6 min), and demonstrated significantly greater sleep fragmentation in week 12 compared to week 1 (*p* = 0.032, 95%CI: 0.22, 7.11, *d* = 0.63, ∆ = 4%) and week 2 (*p* < 0.01, 95%CI: 0.89, 7.16, *d* = 0.72, ∆ = 4%). The proportion of nights per week that recruits did not meet the minimum national sleep recommendations for adolescents (i.e., 8 h) was 100% (week 1), 98% (week 2), 96% (week 6) and 99% (week 12). Average bed and wake-times for JS differed across the reporting weeks of basic training; week 1 (bedtime: 22:46 ± 00:22hm; wake-time: 05:47 ± 00:09hm), week 2 (bedtime: 23:45 ± 00:14hm; wake-time: 05:22 ± 00:11hm), week 6 (bedtime: 22:30 ± 00:16hm; wake-time: 05:31 ± 00:16hm) and week 12 (bedtime: 23:06 ± 00:36hm; wake-time: 05:38 ± 00:10hm).

**Figure 4:**
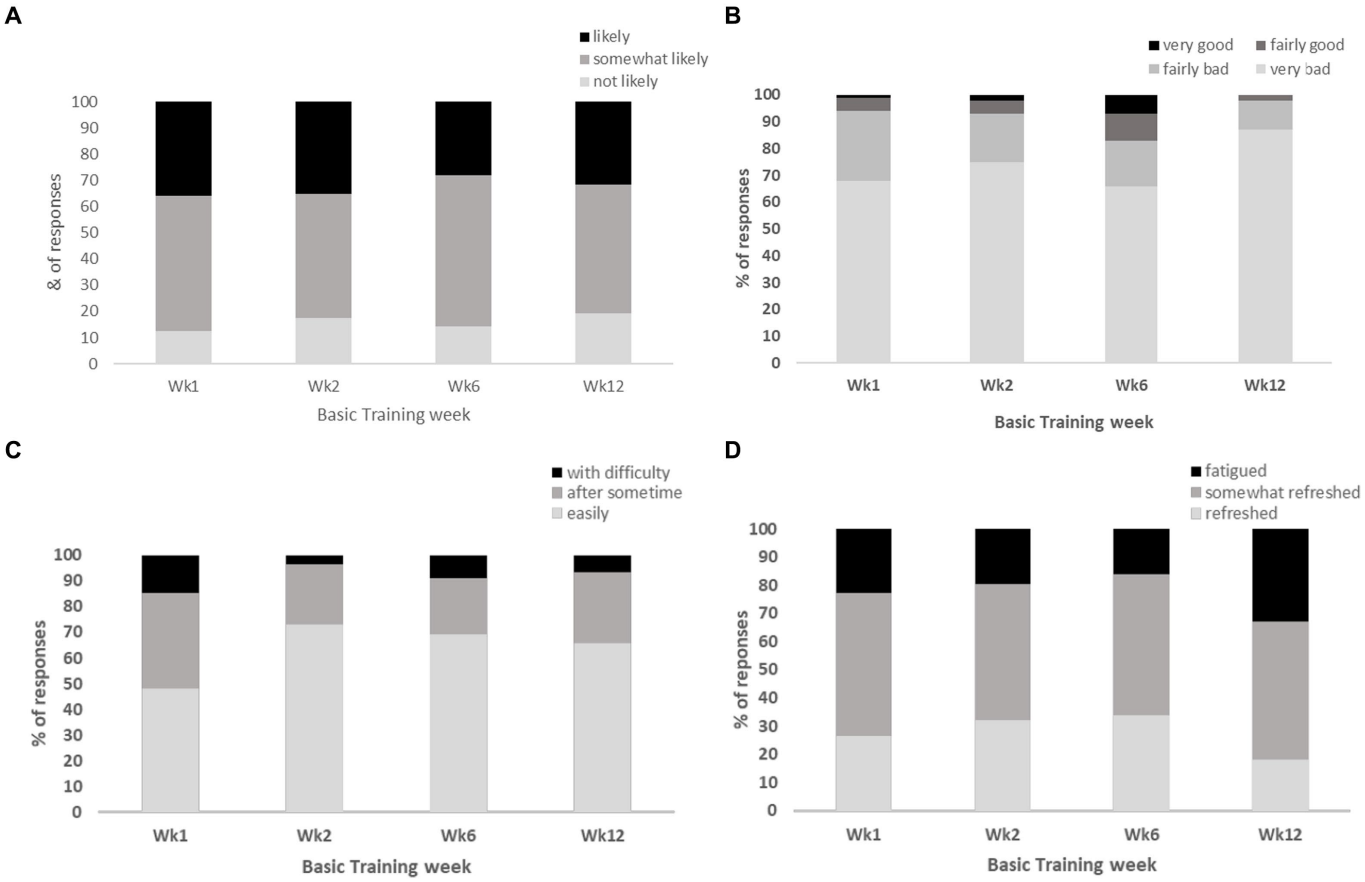
Represents the proportion (%) of responses from JS for the following NSF sleep diary questions, **(A)** “likeliness of dozing off during daytime”; **(B)** “overall rating of sleep quality”; **(C)** “ease of falling asleep at night”; **(D)** “perceived fatigue upon awakening”.

The majority of sleep diary responses across the reporting weeks of basic training indicated that JS were “likely” (28–36%) and “somewhat likely” (47–58%) to doze off during the daytime; they rated their sleep quality as “very bad” (66–87%) and “fairly bad” (11–26%); they were able to fall asleep “easily” (60–83%) and “somewhat easily” (22–37%); and felt “somewhat refreshed” (48–51%) and “fatigued” (16–32%) upon awakening during basic training (Figures 4A–D).

The most common self-reported sleep disturbing factors reported by JS during the reporting weeks of basic training included noise (32–35%) within the primary sleeping environment, early-morning wake times (23–34%), bed discomfort (18–24%), and feelings of stress and anxiety (11–18%).

### Study 2

#### Non infantry recruits

Significant main effects for week of basic training were observed for sleep efficiency (*p* < 0.001, η_p_^2^ = 0.182), TIB (*p* < 0.001, η_p_^2^ = 0.176), TST (*p* < 0.001, η_p_^2^ = 0.188), WASO (*p* < 0.001 η_p_^2^ = 0.227), SOL (*p* < 0.01, η_p_^2^ = 0.171) and sleep fragmentation (*p* = <0.001, η_p_^2^ = 0.087). *Post hoc* analysis revealed significant differences (*p* < 0.05) for all sleep indices between weeks of basic training (Supplementary Table S3), ranging in moderate-to-large effects for significant pairs.

**Figure 5:**
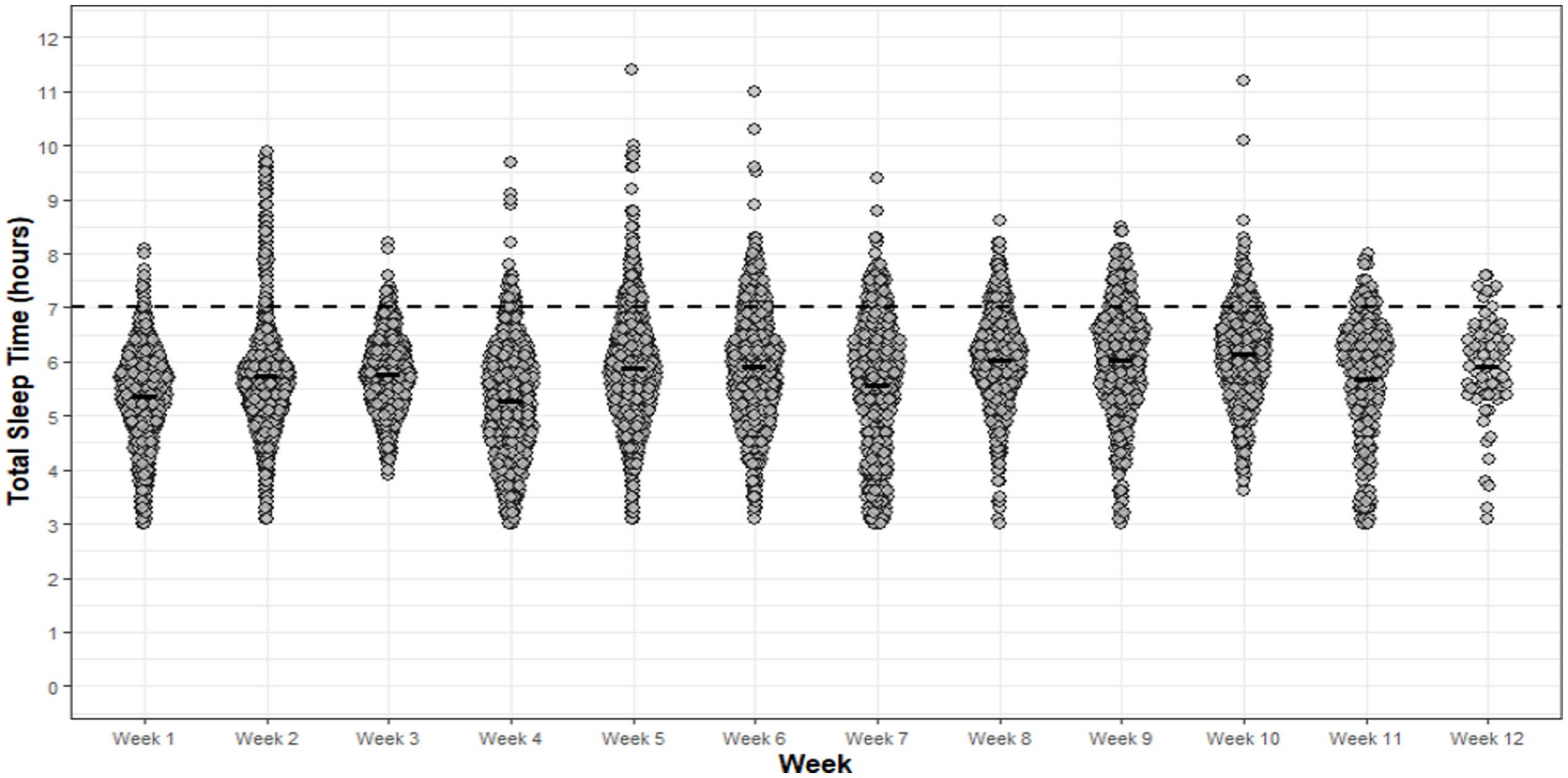
Non-infantry recruit TST achieved for each night of each week of basic training. Dots are individual nights within their respective week and the solid horizontal line represents the average weekly TST. The dashed horizontal line represents the minimum national sleep recommendations for young adults (7 h).

Sleep efficiency was similar across weeks, ranging between 81 and 88%, with the lowest scores observed for week 4. The average SOL across each week ranged from 00:08hm – 00:15hm, with weeks 4, 7 and 12 showing significantly shorter SOL compared to weeks 6, 9 and 10. The shortest average TIB for non-infantry recruits was observed during week 1 (05:53 ± 00:48hm) of basic training. For all other weeks, the average TIB ranged between 06:26 ± 01:15hm and 07:10 ± 00:38hm, with the longest TIB observed in week 10. The shortest average TST was observed in weeks 1, 4, 7 and 11, ranging from 05:09 ± 00:47hm to 05:21 ± 00:47hm. All other weeks demonstrated an average TST of between 05:43 ± 00:40hm and 06:06 ± 00:38hm, with the shortest and longest TST observed for week 1 and week 10 of basic training, respectively (Figure 5). The shortest average WASO was observed during week 1 (00:39 ± 00:15hm), however, WASO ranged between 00:52 ± 00:18hm to 01:18 ± 00:25hm for all remaining weeks of basic training. Sleep fragmentation was found to be greatest during week 12 (36 ± 9.8%), thus indicating greater restlessness, and by extension, sleep disturbance. For all remaining weeks, sleep fragmentation was similar, ranging between 30 ± 7.5% to 35 ± 9.8%. A strong positive correlation between TST and TIB was observed for weeks 1 to 11 of basic training, ranging *r* = 0.681–0.901, *p* < 0.001. Time in bed explained 83–95% of the variance in non-infantry recruit’s TST during basic training. Week 12 showed a weak positive correlation (*r* = 0.239, *p* = 0.034), with TIB. However, explained 49% of the variance in TST.

The proportion of nights for each week that did not achieve the minimum national sleep recommendations for young adults (i.e., 7 h) was 80–98%. Additionally, average bed and wake-times varied between weeks of basic training, ranging from 22:10 ± 02:13hm to 23:55 ± 02:11hm and 04:47 ± 01:52hm to 06:34 ± 01:35hm, respectively.

**Figure 6:**
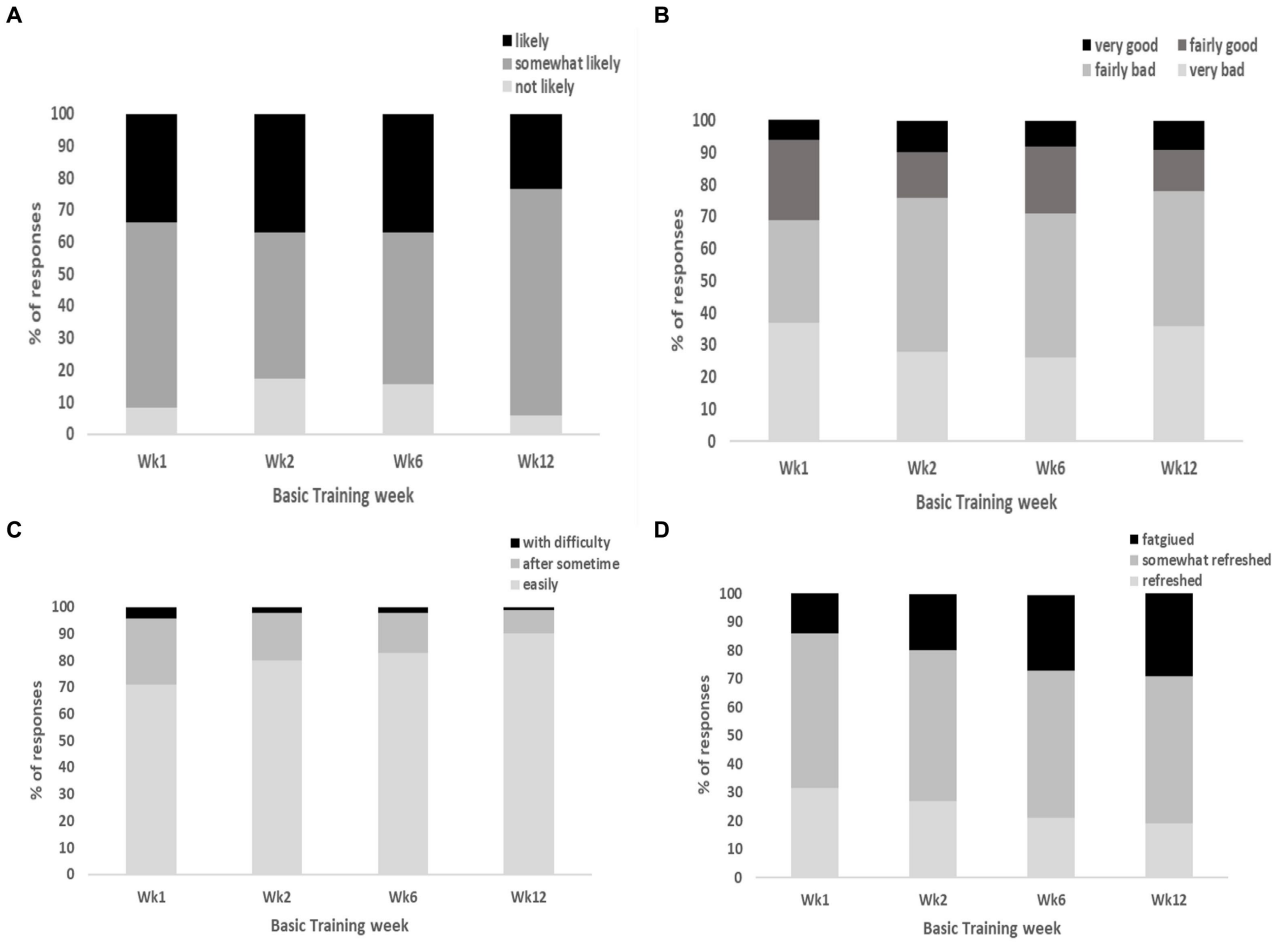
Represents the proportion (%) of responses from non-infantry recruits for the following NSF sleep diary questions, **(A)** “likeliness of dozing off during daytime”; **(B)** “overall rating of sleep quality”; **(C)** “ease of falling asleep at night”; **(D)** “perceived fatigue upon awakening”.

The majority of sleep diary responses across the reporting weeks of basic training indicated that non-infantry recruits were “somewhat likely” (46–71%) and “likely” (24–37%) to doze off during the daytime; they rated their sleep quality as “very bad” (26–37%) and “fairly bad” (32–48%); they were able to fall asleep “easily” (71–90%) and “somewhat easily” (9–25%); and felt “somewhat refreshed” (52–54%) and “fatigued” (19–31%) upon awakening during basic training (Figures 6A–D).

The most common self-reported sleep disturbing factors reported by non-infantry recruits during basic training included late night military admin and early morning wake times (75–80%), noise within the primarily sleeping environment (43–45%), stress and anxiety (18–19%) and muscle soreness (12–14%).

## Discussion

This study sought to evaluate the sleep–wake profiles of British Army JS, infantry and non-infantry recruits, and to quantify modifiable sleep disturbing factors during their respective basic training. Overall, and in agreement with our hypothesis, the average TST observed for JS, non-infantry and infantry recruits was consistently 1-2 h less than the minimum national sleep recommendations. The poor TST can be largely explained by the range in average TIB observed in JS (06:35 ± 00:40–07:04 ± 00:23hm), non-infantry (05:53 ± 00:48–07:10 ± 00:38) and infantry recruits (05:20 ± 00:31hm – 06:54 ± 00:50hm) during basic training. Recruits and JS undertake a number of over-night field-exercises, range activities (i.e., marksmanship) and summative assessments during basic training, most of which are designed to simulate operational demands, which inevitable result in varied sleep schedules. The varied scheduling of training activities explains, in part, the variability in bed and wake times across training for each group. For instance, infantry recruits during week 6 demonstrated ~1 h less average TST compared to other weeks (Figure 6). Upon review of the basic training schedule, it was confirmed that the later bedtimes and shorter TST were due to late-evening foot-drill training in preparation for a summative foot-drill exam scheduled at the end of week 6. Despite the poor average TST for each group across basic training, a small proportion (≤ 32%) achieved minimum sleep recommendations. It is unclear as to how some, over others, were able to achieve minimum sleep recommendations given the standardized nature of training, the unique sleep environment (i.e., 12-person dorms) and the extent of sleep disturbances reported by recruits and JS (i.e., late-night military admin, noise, light etc). However, it is thought that some individuals utilize their time more effectively during the day (when available) to conduct personal military admin (e.g., locker set up, kit cleaning etc.), enabling earlier bedtimes and longer sleep duration. It was also suggested that individuals placed on limited duties due to short-term illness and/or injury may have greater opportunity to achieve longer sleep periods. Nevertheless, further investigation is warranted to elucidate these observations.

Strong positive correlations were observed between TIB and TST for each week of basic training across all three cohorts, indicating that when given greater TIB, most, if not all JS, non-infantry and infantry recruits prioritized a longer TST. Nevertheless, a number of sleep disturbances preventing adequate sleep duration and quality were identified across each cohort, including extraneous light, noise and hot room temperatures within their primary sleeping environment, early-morning wake times and late-night military admin, muscle soreness, bed discomfort and feelings of stress and anxiety. Furthermore, ~12% of JS, non-infantry and infantry recruits described sleeping on the floor next to their bed in fear of failing early-morning locker/kit inspections. The extent of these self-reported sleep disturbing factors are likely key contributors to the average WASO, sleep fragmentation, and reports of “fairly bad” and “very bad” sleep quality during basic training. Based on our observations, we have shown that JS, non-infantry and infantry recruits are achieving inadequate sleep duration with poor (self-reported) sleep quality, impacted by poor sleep hygiene and sleep schedules.

The chronic sleep restriction observed in our populations is similar to that reported in other recruit populations. Larsen et al. (23) and Bulmer et al. (21) conducted objective sleep evaluations of Australian recruits during a 12-week basic combat course and reported an average sleep duration of ~6.4 h per night. It was further reported that a relatively high proportion of recruits (42%) experienced chronic sleep restriction (i.e., <6 h per night on average). Crowley et al. (22) reported similar patterns of chronic sleep restriction in United States Army recruits during basic training who were averaging between 5 and 6 h sleep per night, which recruits reported as having a detrimental effect on their academic performances due to poor concentration, difficulty staying awake and poor retention of key information during class-based activity. As a consequence of restricted sleep, it is well-known that levels of simple (i.e., reaction time, short-memory recall) and complex (i.e., problem solving, critical thinking, learning) cognitive function are significantly improved with sufficient sleep (42, 43). For instance, Andrews (44) reported 11% better standardized academic test scores in US Army recruits that received 8 h sleep per night compared to those receiving 6 h sleep per night, highlighting the importance of sufficient sleep opportunity on academic performances. Sleep restriction has also shown to significantly increase the risk of injury and illness in military and adolescent civilian populations. Grier et al. (10) identified a dose–response relationship between sleep duration and musculoskeletal injury (MSKi) incidence in adult military personnel. Compared to those who slept >8 h per night, military personnel who slept ≤4 h, ≤ 5 h and ≤ 6 h per night were at a 2.35, 2.06- and 1.53-times greater risk of sustaining a MSKi during training, respectively. Similarly, Milewski et al. (24) determined the relationship between sleep and injury within a similarly aged athletic adolescent population and demonstrated that those who slept less than the minimum recommendations (i.e., 8 h per night) were at a 1.7 times greater risk of sustaining a MSKi compared to those achieving >8 h sleep per night. Sleeping <6 h per night during British Army basic training has shown recruits to be at a 3-times greater risk of being diagnosed with a respiratory illness (8), leading to lost training days, re-squadding and potential discharge from basic training. As such, our population are likely to also be at greater risk of injury, illness and overall poorer academic and military specific performances.

In our study, sleep efficiency scores were within normal ranges (45) throughout basic training, suggesting that JS, infantry and non-infantry recruits achieved relatively good sleep despite consistent reports of poor sleep quality and insufficient sleep duration. A possible reason for the discrepancy between the interpretation of sleep efficiency scores (and sleep reports) is likely a result of the short SOL: despite the potential negative health implications, a shorter SOL results in a higher (i.e., better) sleep efficiency score (34). The average SOL for JS, infantry and non-infantry recruits was considered short, ranging between 7 and 14 min. Notwithstanding individual differences, a SOL of ≤30 min is considered normal for a healthy individual, however, a SOL of ≤8 min is considered extremely short, indicating a high degree of sleep pressure due to sleep restriction and/or the existence of an underlying sleep problem (e.g., hypersomnia) (45). Similar to SOL, WASO is a key metric in the determination of sleep efficiency. While current guidelines do not specify a strict threshold (46) a WASO of >20 min may be indicative of poor sleep hygiene and/or an underlying sleep problem (45, 47). The average WASO observed for JS and infantry recruits was ~30 min, whereas non-infantry recruits experienced an average WASO of ~62 min, explaining, in part, the lower sleep efficiency scores compared to JS and infantry recruits. In contrast to Larsen et al. (23), our sleep efficiency scores were up to 14% greater than those reported in Australian Army recruits during basic training. This difference can partly be explained by the similar SOL (range: 16 – 18 min) and greater WASO (range: 85–123 min) observed in the Australian population, reporting lower sleep efficiency scores (< 79%) estimated from similar wearable technology (i.e., GT9X wrist monitor, Actigraph). The short SOL therefore suggests that sleep pressure is high and that recruits are experiencing a high degree of sleepiness which could be related to, and/or result in, an underlying sleep disorder. However, due to the extent of sleep restriction, any potential underlying disorders would be difficult to determine.

Although the majority of JS, infantry and non-infantry recruits fell asleep quickly once in bed, as demonstrated by the short SOL, the average WASO observed across the reporting weeks of basic training indicates a high degree of sleep disturbance after sleep onset. Although short periods of wakefulness (<20 min) after sleep onset are expected as part of the natural sleep–wake cycle (48), the extent of WASO observed while sleeping in their primary accommodation is likely a consequence of the poor sleep hygiene contributing to greater and more fragmented sleep patterns during basic training. The most common sleep disturbances observed are similarly reported by others during training (21–23), including early-morning wake times and late-night military admin, illness, bed discomfort, extraneous light, noise, and hot room temperatures. The use of personal electronic devices (PEDs) was also reported as a factor impeding adequate sleep during basic training, with many taking the opportunity to contact family and friends, many of whom resided oversees with vastly different time zones. Routine sleep disturbance resulting in fragmented sleep has been associated with decreased behavioral alertness and cognitive capacity (i.e., memory consolidation, learning), and increased negative mood changes (49–51). As such, efforts to improve sleep hygiene to enhance sleep quality are therefore warranted, including more comfortable mattresses/bedding, light and noise mitigations, better regulation of (cooler) room temperatures and relevant education to reduce the magnitude of external (i.e., PED usage) sleep disturbances. It is believed that many of the sleep hygiene issues identified in our study stems from the unique environment (albeit common to many training establishments) in which they sleep. The 12-person dorms that are frequently used to conduct early-morning and late-night military admin (e.g., ironing, boot cleaning, kit set up, weapon handling, studying etc.) must therefore be considered in any sleep intervention.

Muscle soreness was reported by JS, infantry and non-infantry recruits as a key factor contributing to disturbed sleep during basic training. Good quality sleep is well-known for its role in optimizing adaptation and recovery of numerous neuro-physiological processes (52–55), and thus, with greater opportunity to recover from the physical stressors of basic training, one would expect reports of muscle soreness, and by extension, sleep disturbances to be reduced (52). Other key sleep disturbing factors included feelings of stress and anxiety. Indeed, reports of depressive symptoms are common during basic training, particularly during initial entry (56). Those entering basic training for the first time, the environment is a stark contrast to their prior home environments (e.g., including routine physical training and class-based lessons/education, communal living quarters, regimented mealtimes and sleep schedules, and chronic sleep restriction). Poor sleep quality due to sleep restriction is associated with higher psychological stress and maladaptive training responses during military training (57–59). For many, the psychological demand of adjusting to the basic training environment coupled with routine sleep restriction is likely to exacerbate the levels of psychological stress experienced during basic training, resulting in potentially lower mood, greater mental health-related problems, and by extension, undesirable attrition rates (22, 56, 59, 60). In some recruits this was compounded through the fear of failing their morning kit inspections, and as a result, conducting military admin (e.g., ironing) late into the evening and sleeping on the floor next to their bed. Simple educational and leadership interventions to reduce these types of counterproductive behaviors during basic training is warranted, alongside further work to understand how to better manage and/or structure the psychological demands of basic training.

Compared to non-infantry and infantry recruits, early-morning wake times were more commonly reported by JS as key sleep disturbing factors, and despite an allotted 8 h sleep period (less field exercise and ranges) during basic training (lights out: 2200, reveille: 0600), the average TST was 06:22hm, with average bed and wake times of 23:02 ± 00:32hm and 05:35 ± 00:10hm, respectively. Despite a growing awareness of adolescent sleep–wake requirements, there remains a common misconception among civilian and military members that adolescents should choose and/or be educated to go to sleep earlier than normal as to improve concentration and prevent sleep loss. However, biological changes in the homeostatic regulation of sleep (i.e., phase delay), which contribute to an increased rise in eveningness-chronotype, leads to extended wakefulness later in the evening (61). Therefore, adolescents will naturally fall asleep ~1-2 h later compared to their adult counterparts (62), which in turn results in later awakenings and demonstrates the futility of earlier bedtimes compared to extended-morning wake/start times on measures of improved physical, behavioral and cognitive function (29, 30, 63, 64). Similar to that of adolescent civilians (30, 55), our study has shown that JS are experiencing chronic sleep restriction throughout basic training, mainly from the interaction between biological adaptation (e.g., puberty, circadian and homeostatic adaptations) and environmental constraints (e.g., early basic training start times, poor sleep hygiene, societal pressures/demands), which is likely to lead to persistent and unrecoverable sleep loss. Failure to account for these biological changes can lead to the development and/or exacerbation of sleep disorders, impaired physical and mental health, increased potential for substance abuse (i.e., drugs, alcohol, smoking), greater risk taking behaviors and negative mood traits, and impaired neurobehavioral function (32, 65). Therefore, it is critical that interventions to improve sleep in JS during basic training (e.g., extended-morning wake times, improved sleep hygiene) must be considered relative to their specific sleep–wake physiology and independent of their adult counterparts.

A number of study limitations must be acknowledged. Our study was only able to evaluate sleep across the initial 12-weeks of JS basic training, and therefore, sleep–wake patterns and disturbances, and perceptions of sleep quality may differ as they progress through their 28-week or 49-week basic training course. Due to resource availability, two types of wearable technology (i.e., Actigraph watch and Oura ring) were used to measure sleep during the two studies, making direct cross-cohort comparisons difficult due to variations in the recorded data between the two technologies. Actigraphy is commonly used due to its levels of acceptability, low cost and utility in monitoring sleep in natural settings. However, actigraphy has shown to under-overestimate certain sleep wake-indices when compared to gold standard (i.e., polysomnography) (66) and therefore, validated wearable technology that incorporates biometric signals (e.g., heart rate) into sleep–wake detection algorithms to improve accuracy are recommended, along with the combined use of sleep diaries. Additionally, no measures of associated outcomes such as sleep disorders, components of psychological distress (i.e., stress, anxiety, depression) or training-related performances were included, and thus preventing a more detailed analysis. Although male and female recruits and JS sleep in single-sex dorms, no sex differences in objective sleep–wake indices were observed for JS or non-infantry recruits during basic training. These findings are likely due to the standardized (gender-free) scheduling and content of basic training. To note, no female infantry recruits were enrolled into basic training at the time of this study. Nevertheless, sex differences in the prevalence of certain sleep disorders (e.g., insomnia) and architecture (67) have been reported, indicating the influence of sex steroids and menstrual cycle on sleep–wake indices (67–69), and therefore, should be considered in subsequent sleep research.

## Conclusion

Our findings demonstrate that sleep restriction is pervasive across basic training, regardless of unit type (i.e., JS, non-infantry and infantry recruits). Despite a growing awareness of the importance of sleep relative to the health, performance and wellbeing of military trainees, it is clear that greater efforts are required (i.e., practical and educational) to both increase sleep duration and improve sleep hygiene practices to reduce the magnitude of sleep disturbances. It is acknowledged that some aspects of basic training (i.e., field exercise) are designed to intentionally restrict sleep as a means of mimicking operational demands. However, based on our observations, sleep restriction is not constrained to field exercise. Rather, the average sleep duration is consistently below minimum sleep recommendations throughout basic training, leading to a high degree of chronic and unrecoverable sleep loss, which in turn is likely to significantly impair physical and cognitive military performance, and increase the risk of injury, illness and greater attrition rates. Changes in the design and scheduling of basic training programs to enable, at the least, minimum sleep recommendations to be met, and to improve sleep hygiene in the primary sleeping environment are warranted.

## Data availability statement

The original contributions presented in the study are included in the article/Supplementary material, further inquiries can be directed to the corresponding author.

## Ethics statement

The studies involving humans were approved by United Kingdom Ministry of Defense Research Ethics Committee. The studies were conducted in accordance with the local legislation and institutional requirements. Written informed consent for participation in this study was provided by the participants’ legal guardians/next of kin.

## Author contributions

AJR: Data curation, Formal analysis, Methodology, Project administration, Supervision, Writing – original draft, Writing – review & editing. HT: Data curation, Project administration, Resources, Writing – review & editing. KH: Validation, Visualization, Writing – review & editing. KJ: Data curation, Project administration, Writing – review & editing. RH: Data curation, Writing – review & editing. SC: Data curation, Methodology, Project administration, Writing – review & editing. AnR: Methodology, Validation, Writing – review & editing.
